# Potential to mitigate ammonia emission from slurry by increasing dietary fermentable fiber through inclusion of tropical byproducts in practical diets for growing pigs

**DOI:** 10.5713/ajas.18.0481

**Published:** 2018-09-13

**Authors:** Quan Hai Nguyen, Phung Dinh Le, Channy Chim, Ngoan Duc Le, Veerle Fievez

**Affiliations:** 1 Laboratory for Animal Nutrition and Animal Product Quality, Faculty of Bioscience Engineering, Ghent University, B-9000 Ghent, Belgium; 2 Faculty of Animal Sciences and Veterinary Medicine, Hue University of Agriculture and Forestry, Hue University, Hue 530000, Vietnam

**Keywords:** Ammonia Emission, Pigs, Fiber-rich Feedstuffs, Manure

## Abstract

**Objective:**

Research was conducted to test the effect of including fiber-rich feedstuffs in practical pig diets on nutrient digestibility, nitrogen balance and ammonia emissions from slurry.

**Methods:**

Three Vietnamese fiber sources were screened, namely cassava leaf meal (CL), cassava root residue (CR), and tofu by-product (TF). Accordingly, a control diet (Con) with 10% of dietary non-starch polysaccharides (NSP) and three test diets including one of the three fiber-rich feedstuffs to reach 15% of NSP were formulated. All formulated diets had the same level of crude protein (CP), *in vitro* ileal protein digestible and metabolisable energy, whereas the *in vitro* hindgut volatile fatty acid (VFA) production of the test diets was 12% to 20% higher than the control diet. Forty growing barrows with initial body weight at 28.6±1.93 kg (mean±standard deviation) were allocated to the four treatments. When pigs reached about 50 kg of body weight, four pigs from each treatment were used for a nitrogen balance trial and ammonia emission assessment, the remaining six pigs continued the second period of the feeding trial.

**Results:**

The TF treatment increased fecal VFA by 33% as compared with the control treatment (p = 0.07), suggesting stimulation of the hindgut fermentation. However, urinary N was not significantly reduced or shifted to fecal N, nor was slurry pH decreased. Accordingly, ammonia emissions were not mitigated. CR and CL treatments failed to enhance *in vivo* hindgut fermentation, as assessed by fecal VFA and purine bases. On the contrary, the reduction of CP digestibility in the CL treatment enhanced ammonia emissions from slurry.

**Conclusion:**

Dietary inclusion of cassava and tofu byproducts through an increase of dietary NSP from 10% to 15% might stimulate fecal VFA excretion but this does not guarantee a reduction in ammonia emissions from slurry, while its interaction with protein digestibility even might enhance enhanced ammonia emission.

## INTRODUCTION

Ammonia is one of the forms through which nitrogen can be lost from animal production units and might contribute to acidification and eutrophication of the environment [1]. Moreover, ammonia contributes to the formation of fine particles which are harmful to human health [2].

A nutritional strategy to mitigate ammonia emission from pig production systems is to change the fermentable dietary fiber type and/or increase fermentable dietary fiber levels in isoproteic diets to i) induce a shift of N excretion from urine to feces and ii) to reduce slurry pH [3,4]. Indeed, the main part of urinary nitrogen is lost as urea which is rapidly converted to ammonia when exposed to fecal urease. Urinary nitrogen excretion is hypothesized to be partially shifted to bacterial protein in feces by including fibrous feedstuffs in the diet when stimulating the fermentation in the hindgut [4]. To meet the microbial N requirements under circumstances of enhanced hindgut fermentation, transfer of blood urea-N to the hindgut could be upregulated at the expense of transfer to the kidney [5]. Alternatively, sequestration of NH_3_ from protein degradation in the hindgut might be improved [5]. Accordingly, shifting nitrogen excretion from the urine to feces possibly might reduce ammonia volatilization [4]. Moreover, stimulation of fecal volatile fatty acid (VFA) production in the slurry of pigs fed fiber-rich diets might decrease slurry pH [4,6] which plays a crucial role in controlling ammonia volatilization from animal slurry [7].

However, fibers in various feed resources largely differ in physical structure and chemical characteristics. This not only influences their potential to reduce ammonia losses, but also their interaction with other nutrients which might reduce digestibility and absorption of nutrients [8]. In this respect, fiber might affect ileal protein digestibility by modifying the viscosity of digesta [9], which obviously might impair animal performance.

Three fibrous feed resources varying in total and proportional soluble and insoluble fiber content as well as *in vitro* stimulation of hindgut fermentation (Table 1) were selected for the current *in vivo* evaluation, i.e. cassava leaf meal (CL), cassava root residue (CR), and tofu by-product (TF).

The research was conducted as a screening test to assess these fiber sources for their potential to reduce ammonia emissions. Fermentable fiber is hypothesized to reduce ammonia emissions from pig slurry by a stimulation of the hindgut fermentation, shifting the rather volatile urinary nitrogen excretion towards a more stable form of fecal nitrogen and reducing the slurry pH. Moreover, dietary fiber also is hypothesized to interact with other nutrients resulting in a reduction of nutrient digestibility, especially digestibility of crude protein (CP). For this purpose, each of the three sources were included in a practically feasible diet to assess the effect on nitrogen balance, urinary and fecal composition as well as ammonia emission from pig slurry.

## MATERIALS AND METHODS

### Animal care

Animal housing and experimental procedures were in accordance with the experiment performed by Tanghe et al [10] according to the recommendations by the Ethical Committee of ILVO (approval number 2010/123).

### Animals and housing

The experiment was conducted at Hue University of Agriculture and Forestry in Thua Thien Hue Province (16°00′ to 16°48′ latitude, 107°48′ to 108°12′ longitude) in Vietnam. Forty growing Duroc×(Landrace×Yorkshire) crossbred barrows with initial body weight (BW) of 28.6±1.93 kg (mean±standard deviation) were used for the experiment. They were individually housed in pens of 2 by 0.75 m with concrete floor, had access to separate feeding troughs and free access to fresh drinking water.

### Experimental design

Pigs were allocated to the four treatment groups to equally stratify all groups in terms of BW and were given a two-week adaptation period to the four experimental diets (ten pigs per treatment). The treatments included a control diet (Con) of which the formulation was based on common ingredients used in commercial diets for growing pigs [11]. Feed ingredients included in the control diet are presented in Table 2. Diets were formulated based on recommendations for metabolisable energy (ME) content according to NRC [12] and contained 18% and 15% CP for the first (until 50 kg BW) and second (50 to 80 kg BW) growing phase (Tables 2, 3), respectively. Further, recommendations for dietary lysine content (9.5 g and 7.5 g lysine/kg for the first and second growing phase, respectively) according to NRC [12] were respected and ileal digestible protein content (assessed through *in vitro* simulation as explained in 2.6.) was kept constant at 140 and 120 g/kg in the first and second growing phase, respectively. Non-starch polysaccharide (NSP) content of the Con was fixed at 10%. The test diets were indicated as CL (diet with cassava leaf meal as fiber-rich feedstuff), CR (diet with cassava root residue as fiber-rich feedstuff), and TF (diet with tofu by-product as fiber-rich feedstuff).

The test product included in CR refers to the solid fibrous residue that remains after the starch content has been extracted from cassava roots, whereas cassava leaves left after harvesting the roots were included in the CL diet. They were collected at harvest, sundried and ground. Finally, the TF was collected from the following process: soybeans were dehulled, ground and soaked into water. Next the solution was centrifuged to recover the soy milk. The solid residue from this step was the fresh TF, which then was sundried. These feedstuffs differed in total NSP and proportion of soluble and insoluble fiber (Table 1), as well as in fermentative capacity as assessed through production of VFA during an *in vitro* hindgut simulation set-up. An outline of the *in vitro* set-up is given.

The CL, CR, and TF diets contained 15% NSP in which the three fiber-rich feedstuffs each contributed for 15% to the total dietary NSP content (Tables 2, 3). The feeding trial was divided into two periods; a first period, for pigs with a BW from 28 to 50 kg, lasted 31 days and a second period, for pigs with BW of 50 to 80 kg BW, lasted 37 days.

When pigs reached an average BW of 50 kg, pigs in each treatment were ranked from low to high BW and pigs ranked 1st, 4th, 7th, and 10th were selected for the N balance and ammonia emission trial, the remaining six pigs continued the second period of the feeding trial.

Prior to the nitrogen balance and ammonia emission trial, the 16 selected pigs were offered the diets of the second period during one week before they were moved to the metabolism cages (1.2 m×0.6 m; length×width) where they were housed individually. They were allowed to adapt to the new environment for two days after which the experiment started and lasted for seven days; including three days for separate collection of urine and feces to be used in the laboratory ammonia assessment set-up and the last four days for nitrogen balance samplings. No change in feed intake nor behavior was observed between period one and two or during the period in the metabolism cages.

In both periods as well as in the feeding trial and the nitrogen balance trial, pigs were fed three equal daily meals at 8:00, 14:00 and 20:00. Feed supply was adjusted daily and offerings were 10% higher than the feed intake of the previous day. Feed residues were removed daily. Feed offer and refusal were recorded daily. The BW of animals was measured monthly to calculate average daily gain (ADG).

### Diet formulation

Through the dietary inclusion of the test feeds, the current *in vivo* screening aimed to evaluate their effect on ammonia emission from manure through formulation of practically applicable diets.

Practical applicability implies the risk to affect animal performance was minimized. Accordingly, diets were formulated to reach similar nutritive values. For this purpose, diets were formulated using the Solver application in Excel to target a NSP level of 10% for the Con diet and 15% for three test diets. Moreover, all diets were formulated to meet following additional constraints. For the first growing phase (28 to 50 kg BW): CP content between 17.7% and 18.2%, *in vitro* ileal digestible CP between 140 and 145 g/kg, ME content of 15.2 MJ/kg and a contribution of NSP from the fibrous feed resources to the total dietary NSP of 15%. For the second growing phase (50 to 80 kg BW); dietary characteristics were as follows: CP between 14.7% and 15.3%, *in vitro* ileal digestible CP between 120 and 125 g/kg, ME content of 15.2 MJ/kg and a contribution of NSP from the fibrous feed resources to the total dietary NSP of 15%.

Given the large variation in chemical composition and nutritive values of the test sources, diets only could be formulated within relatively narrow ranges, i.e. practically impossible to increase the NSP content above 15% while keeping the other characteristics, as mentioned above, equal.

### Collecting samples

#### Ammonia assessment

Urine and feces were collected separately and weighed daily during a 3-day period. Urine collected for further ammonia assessment was not acidified. Containers with the separate urine and feces collections were placed in styrofoam boxes with ice to avoid ammonia volatilisation. After 24 h collections, urine and feces were stored at −20°C of the 3-day collecting period. Afterwards, all frozen urine and feces from each pig were thawed and mixed thoroughly to make a slurry which was used for the ammonia emission assessment.

Ammonia emission was measured in a laboratory system according to the procedure described by Canh et al [13]. Briefly, a sample of three kg slurry per pig was placed in a vessel with an inside area of 284 cm^2^ covered by a lid. Air entered the vessel by small holes on the edge of the lid and left the vessel in the centre. Ammonia in the outgoing air was trapped by passing through two impingers (glass bottles), each containing 100 mL HNO_3_ (0.5 M). The second impinger served as a control and contained no more than 5% of the amount of ammonia trapped in the first impinger. The air left the system after passing through the impingers. The flow rate was adjusted to 1 L/min through a capillary controller.

The two impingers were replaced after 1, 2, 4, and 7 days. The ammonia concentration and the volume of the liquid were determined in the first and the second impinger. The total ammonia emission from each pig (vessel) was calculated by multiplying the total volume of acid in each impinger by the ammonia concentration.

At the same time, another three kg of slurry from each pig was run in a parallel system without ammonia trapping to measure pH variation over the same time. The slurry pH was measured at 6 cm depth layers of the slurry at the starting day (day 0) as well as day 1, 2, 4, and 7.

As the laboratory capacity was limited, four slurry samples from four treatments were measured for ammonia at the same time (in one week). Hence, the ammonia emission assessments were performed over a period of four weeks.

#### Sample collection to determine nitrogen balance

Total urine and feces were collected separately and weighed every morning. Urine was collected by a urinary tray which was connected to the basket by a tube. As an extra precaution to avoid nitrogen loss due to ammonia volatilization, H_2_SO_4_ (50 mL, 25%) was added to the tray prior to the urine collection to keep the pH below two. About 20% of the total urine and feces were taken as sub-samples daily and stored at −20°C. After the four collection days, frozen samples of urine and feces from each pig were thawed, mixed and duplicate sub-samples of urine and feces from each pig were taken for further analyses. Sub-samples of feces required for further analyses were dried at 50°C.

### Chemical analyses of feeds, feces, and slurry

The dry matter (DM) contents of feed ingredients, composed diets, feces, and slurry were determined by drying at 105°C for 16 to 20 h until constant weight [14]. Ash content of feed ingredients, composed diets, feces, and slurry were determined by drying at 550°C for 3 to 5 h [14]. Nitrogen concentration in the feed ingredients, composed diets, urine and feces was determined using the Automatic Kjeldahl destruction and distillation machine (Velp Scientifica, Usmate, Italy) following the Kjeldahl method [14]. Feed ingredients, composed diets and feces were analysed for ether extract (EE) using the Soxhlet method (Sci Finetech, Seoul, Korea) [14]. Neutral detergent fiber was analysed including heat stable amylase and expressed inclusive of residual ash. Acid detergent fiber (ADF) expressed inclusive of residual ash and acid detergent lignin (ADL) was determined by solubilization of cellulose with sulphuric acid. Both parameters were analysed following the protocol of the Ankom Company, using the Ankom A200 (USA) in the feed ingredients.

The insoluble fiber, soluble fiber and total dietary fiber of feed ingredients were analysed according to a protocol described by Prosky et al [15]. “Briefly, about 1 gram of sample was weighed in an incubation flask to which 40 mL of a solution containing 9.76 g/L 2(N-morpholino) ethanesulfonic acid (Sigma-Aldrich, Diegem, Belgium); and 6.1 g/L 2-amino-2-(hydroxymethyl)-1,3-propanediol (Sigma-Aldrich, Belgium); as well as 50 μL heat-stable alpha-amylase solution (Special quality for TDF assays, Megazyme Inc., Chicago, IL, USA) was added. The incubation flask was put in a boiling waterbath with continuous agitation for 30 min. Afterwards a 100 μL protease solution (Special quality for TDF assays, Megazyme Inc., USA) was added to each sample which was kept in a shaking water bath at 60°C for 30 min. Prior to another incubation of 30 min at 60°C in a shaking water bath, 200 μL amyloglucosidase solution (Special quality for TDF assays, Megazyme Inc., USA) was added. Then, the solution was filtered through a P2 filtration crucible (Pore size 15–90 μm, ROBU Glasfilter-Geraete GmbH Schützenstraße, Hattert, Germany) for fiber extraction, the residue from P2 crucible was then analysed for DM, CP, and ash. From the results of the P2 crucible residue, insoluble fiber was calculated as: DM – CP – ash. To the filtrate, boiling ethanol was added, and this mixture was then filtered through a P3 filtration crucible (Pore size 15 to 40 μm, ROBU Glasfilter-Geraete GmbH Schützenstraße, Germany) for fiber extraction. The residue from P3 crucible was analysed for DM and ash. From the results of the P3 crucible residue, soluble fiber was calculated as: DM – ash. Total dietary fiber was the sum of soluble fire and insoluble fiber content.

The sugar and starch content of feed ingredients were measured using phenol-sulfuric acid according to a protocol of Chow and Landhausser [16]. Ammonia emissions trapped in HNO_3_ from the laboratory slurry set-up was analysed using the technique described by Chaney and Marbach [17]. Briefly, 100 μL of sample taken from the first and second impringer were transferred to an experimental tube. Next, 4.5 mL of a solution containing 10 g/L phenol (Merck, Darmstadt, Germany) and 0.05 g/L sodium nitroprusside dihydrate (Na_2_ [Fe{CN}_5_NO]·2H_2_O; Merck, Germany), as well as 4.5 mL of a solution containing 5 g/L sodium hydroxide (NaOH) and 4.2 mL/L 10% sodium hypochlorite (NaClO) (Merck, Germany) were added, vortexed and left at room temperature for 4 h. Ammonia was quantified using an external standard (1 to 200 mg/L ammonium chloride [NH_4_Cl] Merck, Germany) and by measuring the absorbance at 625 nm.

The VFAs of feces were analysed using a chromatographic method as described by Wambacq et al [18], with GC specifications as described by Gadeyne et al [19]. The purine bases of feces were analysed according to Martinez-Puig et al [20], with high performance liquid chromatography specifications as described by Vlaeminck et al [21].

The NSP in feed ingredients were calculated as DM – crude ash – CP – EE – starch – (Sugar×0.965) – ADL [22].

### *In vitro* assessment of ileal digestibility of crude protein and fermentative capacity in the hindgut of individual feed ingredients

The *in vivo* experiment aimed to screen three feed resources with varying capacity to enhance hindgut fermentation (Table 1) as assessed through *in vitro* simulation following the protocol described by Vervaeke et al [23]. Details on composition of the buffer can be found in the original publication, here an outline of the *in vitro* simulation is given, including three steps simulating digestion in the stomach, small intestine and hindgut, respectively.

At the start of the *in vitro* assessment, two g of finely ground samples (<1 mm) were weighed in Erlenmeyer flasks to which 30 mL HCl 0.075 M was added where after the pH was adjusted to two with 1 M HCl or 1 M NaOH solution. Then, 10 mL of 0.8% pepsin (Porcine, 2000 FIP-U/g, Merck No.7190) in 0.075 M HCl was added. Flasks were closed with parafilm and placed in a shaking incubator at 39°C for 2 h. At the start of step two, simulating luminal digestion in the small intestine, pH in each flask was adjusted to pH 7.5. Then, 40 mL of the pre-warmed and freshly prepared 1% pancreatin solution (10 g pancreatin in one litre of phosphate buffer) at pH 7.5 was added to each flask. Flasks were closed with parafilm and placed in a shaking incubator at 39°C for 4 h. Then, the incubation content was quantitatively recovered in a glass centrifugation tube (empty weight of the centrifugation tube was registered) which was centrifuged at 2,000 g for 10 minutes. The supernatant was removed by pouring the glass tube via a nylon cloth (pore size of 37 μm). Afterwards, 20 mL distilled water was added to the Erlenmeyer to wash the pellet. The washed pellet was recovered after centrifugation (2,000 g, 10 minutes). This washing step was repeated a second time. The residue in the glass centrifugation tubes and nylon cloth were put in the oven at 60°C for 48 h and weighed (after cooling in the desiccator). The first two steps were repeated four times to generate enough residue to be pooled and used as substrate for the fermentation step (step 3). Additionally, analysis of the CP content of the pooled residue allowed to calculate the amount of CP digestible in the small intestine.

Fecal inoculum was used in the third step. Feces from five pregnant donor sows were collected directly from the rectum, weighed and mixed in equal portions in a beaker under permanent flux of CO_2_. Phosphate-bicarbonate buffer was added and the mixture was homogenized with a hand blender for one minute. The mixture then was filtered through cheese cloth and diluted with a phosphate-bicarbonate buffer to reach a concentration of 0.05 g feces/mL buffer.

Substrate in step three (hindgut simulation) consisted of 200 mg of dried residue of step two, which was added to an incubation flask (volume of 120 mL) and wetted with one mL of distilled water. Afterwards, the flask was closed with a rubber stopper and aluminium cap and attached to a vacuum system. The air was removed with the vacuum pump and the flask was filled with CO_2_. Then, 19 mL of prewarmed phosphate-bicarbonate buffer solution mixed with fecal inoculum was added to the flask which was then placed in the incubator at 39°C under frequent and regular shaking for 48 h. After the incubation, VFAs were measured in the supernatant after centrifugation (22,000 g, 15 minute) according to Gadeyne et al [19]. Three statistical replicates (using different sow fecal inocula) were done for each ingredient. All ingredients used in the experimental diets were exposed to such 3-step *in vitro* assessment which allowed to calculate intestinal digestibility of the CP and hindgut VFA production for each of the as well as all experimental diets (Tables 2, 3).

### Statistical analyses

Effects of dietary treatments on growth performance, feces characteristics, nitrogen balance, digestibility and fecal concentration of purine bases and VFAs of pigs were analysed using analysis of variance (ANOVA) according to model 1 and a post-hoc Dunnett test by SPSS (version 24.0) to compare the difference between each treatment and the control when the p value of the ANOVA <0.05.

The individual pig was the experimental unit in model 1, according to:

(1) $${\text{Y}}_{\text{ij}}=\mu +\alpha_{\text{i}}+{\text{e}}_{\text{ij}}$$

Where Y _ij_ = dependent variable; μ = the overall mean; α_i_ = the fixed effect of treatment (i = Con; CL, TF, CR); e_ij_ = the random error.

Ammonia emission and slurry pH were treated using a mixed model for repeated measures procedure in SPSS (version 24.0). The effect of sampling time was evaluated as a fixed repeated measure using the autoregressive moving average model as covariance structure based on the Akaike’s information criterion with pig within period as subject. Further, treatment was considered as a fixed effect and block as a random factor.

## RESULTS

### Animal performance and nutrient digestibility

Table 4 reports animal BW, ADG, feed intake and feed conversion ratio (FCR) in the two periods. In the first period, feed intake of pigs fed the TF treatment tended to be higher than that of the Con treatment (p<0.1). There was no difference on ADG between the Con treatment and the test treatments. Pigs on the CL treatment tended to show a higher FCR than pigs on the Con treatment (p<0.1).

During the second period, pigs on the CR treatment reached a higher final BW and hence had a greater ADG than pigs on the Con treatment (p<0.05). During this period, feed intake was not affected. Hence, the FCR of pigs on the CR treatment was lower than pigs on the Con treatment (p<0.05).

The total apparent digestibility of various chemical fractions is presented in Table 5. For all organic fractions, the CL diet showed a lower digestibility than those of the Con diet (p< 0.05). The DM digestibility of the CR treatment tended to be higher than that of the Con treatment (p<0.1).

### Nitrogen balance, urine, and fecal characteristics

Nitrogen balance parameters and fecal concentration of VFAs and purine bases are shown in Table 6. The nitrogen balance and fecal purine bases concentrations of test treatments were not different from those of the Con treatment.

Feces of pigs on the TF treatment contained or tended to contain higher concentrations of total VFA, particularly butyrate and valerate, than feces of pigs on the Con treatment (p<0.1). The CR treatment resulted in higher fecal iso-butyrate and iso-valerate concentrations than the Con treatment (p<0.01).

### Slurry pH and ammonia accumulation

**Figure 1:**
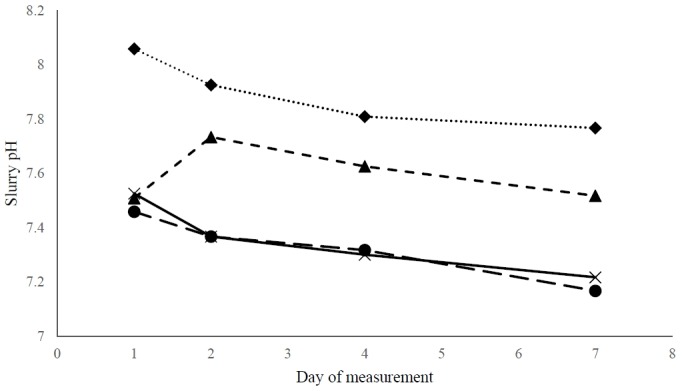
Change in pH of slurry over a 7-day *in vitro* assessment with an equivalent of slurry produced during one day by pigs fed either a control diet (Con) (solid line) or a diet in which cassava root residue (CR) (dotted line), tofu by-product (TF) (long-dash line), or cassava leaves meal (CL) (short-dash line) were included (N = 4 per treatment). Standard error of the mean = 0.18, P_treatment_ = 0.15, P_day_ = 0.15, P_treatment×day_ = 0.60.

The variation in slurry pH throughout the 7-day measurement period is illustrated in Figure 1. Slurry pH did not differ between treatments (p = 0.18). All treatments showed a gradual decrease in slurry pH from day 1 to day 7, except for the CL treatment where the pH decrease was preceded by an initial increase during the first two days.

**Figure 2:**
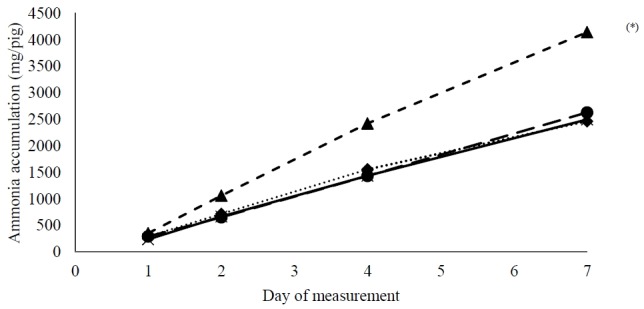
Accumulated ammonia emission over a 7-day measurement period using an equivalent of slurry produced during one day by pigs fed either a control diet (Con) (solid line) or a diet in which cassava root residue (CR) (dotted line), tofu by-product (TF) (long-dash line), or cassava leaves meal (CL) (short-dash line) were included, ^(*)^ indicates a tendency (P_Dunnett_ = 0.01) of CL to differ from the Con treatment, (N = 4 per treatment). Standard error of the mean = 305, P_treatment_ = 0.26, P_day_<0.001, P_treatment×day_ = 0.035.

The effect of dietary treatments on the accumulated ammonia emission (mg/pig) over the 7-day period is illustrated in Figure 2. Globally, over the four measurement days, ammonia emission from the CL slurry tended to be higher than the control (P_CL vs Con_ = 0.10). This difference became more outspoken from day two onwards, as reflected in the interaction effect between treatment and time (p = 0.035) and exceeded the emissions of the other three treatments by 60% and 70% on day four and day seven, respectively.

## DISCUSSION

Fermentable fiber was hypothesized to stimulate hindgut fermentation, potentially reducing the slurry pH and shifting the rather volatile urinary nitrogen excretion towards a more stable form of fecal nitrogen. However, such effects might be obvious only after some time as adaptation of pigs to dietary fiber digestion is suggested to be a long process which requires up to 4 to 5 weeks [24]. Accordingly, nitrogen balance, urinary and fecal composition as well as ammonia emission from pig slurry was studied in Phase 2 of the experiment, i.e. 40 days after dietary inclusion of the fibrous test products. Although not the main focus of the current experiment, growth performance and feed conversion data were reported for Phase 1 (28 to 50 kg BW) and Phase 2 (50 to 80 kg BW) (Table 4). Given the limited number of animals (N = 10 in Phase 1 and N = 6 in Phase 2) as well as the limited period of exposure to dietary fiber in Phase 1 (31 days), these data should be handled with care.

Isoproteic diets were formulated which also contained a fixed content of ME as well as ileal digestible CP, while the NSP content was increased from 10% to 15%. Increased amounts of hemicellulose (ADF minus ADL) accounted for 30% to 40% of the difference in NSP content (Tables 2, 3). Moreover, the tests diets contained more fermentable organic matter than the control diet as 12% to 20% more VFA were produced during an *in vitro* simulation of the hindgut fermentation (Tables 2, 3).

Also *in vivo*, the TF treatment tended (p<0.07) to increase fecal VFA as compared to the control treatment, particularly butyrate (p<0.05) and valerate (p<0.1). Greater fecal VFA concentrations are supposed to be indicative of a stimulation of the hindgut fermentation as observed in the study by Hansen et al [25] for the diet including fiber as compared to the control. However, Canh et al [4] found no significant effect in fecal VFA between a treatment that included 300 g/kg of sugar beet pulp and a control treatment. This might be due to the absorption of most of the VFA in the caecum and large intestine [26], particularly in case of soluble fiber which is rapidly fermented in the proximal colon. On the other hand, insoluble fiber is slowly fermented throughout the large intestine [27] and might contribute to fecal VFA. Soluble fiber only represented a minor fraction of the total dietary fiber in the three test feeds, while in the complete diets soluble fiber varied between 13% and 16% of the total fiber (Tables 2, 3) without difference between dietary treatments. Accordingly, fecal VFA is supposed to be a valid indicator to compare differences in hindgut fermentation in the current study. As such, fecal VFA concentration of the TF treatment increased by 33% (p = 0.07) compared with the Con treatment, while purine bases numerically also increased by 13% (p = 0.49). Both metrics suggest enhanced microbial fermentation in the hindgut for the TF treatment. This *in vivo* observation might be surprising as dietary fiber characteristics hardly differ between the Con and the TF treatment but is in line with results of the *in vitro* assessment. Accordingly, *in vitro* assessments might be more appropriate to evaluate the potential of *in vivo* hindgut stimulation than the chemical fiber characteristics determined as applied here. Moreover, fecal VFA has been suggested to determine slurry pH [7], which besides urinary urea concentration and temperature is a major determinant of ammonia emissions from slurry [28]. However, slurry pH of the Con and TF treatment did not differ, although fecal VFA concentration of the Con treatment only represented about 75% of the TF treatment. Nevertheless, slurry pH also is determined by other factors such as electrolyte balance [13] besides fecal VFA. Additionally, the apparent stimulation of the hindgut fermentation, induced by the TF treatment, failed to reduce urinary N excretion through a shift to fecal N. The urinary N to fecal N ratio was used as an indicator to assess this shift but did not differ between the control and TF treatment (P_Dunnett Con vs TF_ = 0.129). Given the minor changes in urinary N, urinary N/fecal N and the similar slurry pH of control and TF, it is no surprise that this strategy failed to reduce ammonia emissions.

The other two test diets did not enhance fecal VFA and presumably failed to stimulate hindgut fermentation as compared with the control. Moreover, the CL treatment even tended to enhance ammonia emissions (p = 0.10; Figure 2). This potentially originated from the lower CP digestibility associated with this treatment (p<0.01; Table 5). Most probably, dietary inclusion of CL reduced protein digestibility at the level of the small intestine. Unfortunately, it was impossible with the current experimental set-up to assess digestibility in the small intestine. The reduced digestibility might have been caused by an interaction of the dietary fiber with the digestibility in the small intestine. Dietary fiber can reduce digestibility of most nutrients [3,8], an effect which is highly dependent on the physico-chemical characteristics of the fiber [8]. This interaction could change the viscosity of digesta resulting in changes in passage rate and finally affecting the digestibility of nutrients. Particularly protein digestibility might be affected, as reviewed before, but results of various studies are equivocal [9]. Moreover, the CL diet of the current study also contained a higher amount of ADL (Tables 2, 3). This indigestible fraction also could have enhanced endogenous protein losses from the small intestine [29], resulting in reduced apparent CP digestibility. Additionally, hydrogen cyanide (HCN) in cassava leaves has been reported as an important toxin for animals. Indeed, in fresh leaves its concentration can rise up to 400 mg/kg DM [30]. However, it is unlikely that this might have caused the reduction in digestibility as observed here as the leaves used in the diet were dried and drying has been found to be effective in reducing the HCN content [30]. Only CL differed from the three other treatments, including CR, although CL and CR are both byproducts from the cassava plant. However, the chemical characteristics, of the plant parts (i.e. roots and leaves) from which both byproducts (CR and CL, respectively) originate are quite different which resulted in two distinct products (Table 1).

Overall, differences in fermentable fiber of the current diets did not allow to mitigate ammonia emissions compared to the control treatment, which quantitatively were in line with other findings (420 mg/pig/d) [31]. On the contrary, caution should be taken when including fibrous ingredients, as interaction with CP digestibility even might enhance ammonia emissions. Future studies could consider testing higher inclusion levels of fibrous sources with minor effects on digestibility (e.g. TF and CR) as others (e.g. Canh et al [28]) found significant effects on ammonia emissions when supplementing up to 300 g/kg of sugar beet pulp in a diet which induced a rise in total dietary NSP content to 380 g/kg DM compared with control diets with a total dietary NSP content between 130 and 180 g/kg DM. In future studies, it also might be of interest to monitor other effects as inclusion of fiber in pigs’ diet not only has received attention in recent years as a possible environmental mitigation strategy but also because (some) fibers have beneficial effects on animal welfare and behavior, gut health as well as intestinal microflora [32].

## CONCLUSION

Modest dietary inclusion in practical pig diets of cassava or tofu byproducts to increase fermentable fiber might stimulate fecal VFA excretion but this does not guarantee a reduction in ammonia emissions from slurry, while its interaction with protein digestibility even might enhance the risk of enhanced ammonia emissions.

## Figures and Tables

**Table 1:** Chemical composition of the three fiber-rich resources used in the experiment

Variable	CL1)	TF1)	CR1)
Dry matter (%)	93.3	92.8	88.1
Crude protein (%)	27.0	19.1	1.35
Ether extract (%)	9.00	6.50	0.55
NDF (%)	36.0	45.0	ND2)
ADF (%)	26.0	32.3	24.3
ADL (%)	11.0	2.10	3.24
Ash (%)	7.60	4.00	1.90
Starch (%)	8.01	3.98	56.7
Sugar (%)	0.71	0.31	7.70
Insoluble fiber (%)	39.9	62.7	29.3
Soluble fiber (%)	3.30	5.16	5.83
Total dietary fiber (%)	43.2	67.8	35.1
NSP (%)	30.1	56.3	24.3
Total VFA (μmol/g)	2.44	7.73	4.36

NDF, neutral detergent fiber; ADF, acid detergent fiber; ADL, acid detergent lignin; NSP, non-starch polysaccharide; VAF, volatile fatty acid.

1) CL, cassava leaves meal; TF, tofu by-product; CR, cassava root residue.

2) ND, not determined, unreliable NDF result due to the high amount of starch which was incompletely removed by heat-stable amylase.

**Table 2:** Feed ingredients and chemical composition of the experimental diets in the first period (28 to 50 kg of body weight)

Items	Diet1)			

	Con	CL	TF	CR
Ingredient (%)				
Test ingredient2)	-	7.49	3.99	9.26
Finely ground corn	26.0	20.0	18.9	15.0
Rice bran	39.5	22.7	23.1	18.5
Cassava powder3)	15.9	29.4	33.0	33.3
Soybean meal	11.6	17.7	12.6	15.5
Fish meal	6.90	2.70	8.50	8.50
Soybean oil	2.40	2.30	2.00	2.60
L-lysine4)	0.10	0.15	0.06	0.02
Chemical composition				
Dry matter (%)	92.6	94.4	93.0	93.0
Crude protein (%)	17.9	18.2	17.9	17. 7
Metabolisable energy (MJ/kg)	15.2	15.2	15.2	15.2
Ash (%)	5.22	4.92	5.23	4.94
NSP (%)	10.0	15.0	15.0	15.0
IDCP (g/kg)	141	143	141	143
NDF (%)	20.3	19.7	18.7	ND5)
ADF (%)	6.98	8.88	8.30	8.97
ADL (%)	1.60	2.22	1.52	1.60
Starch (%)	45.1	42.7	43.1	44.1
Insoluble fiber (%)	19.5	18.8	18.4	17.5
Soluble fiber (%)	2.99	3.13	3.07	3.34
Total dietary fiber (%)	22.5	21.9	21.5	20.8
Total VFA (μmol/g)	3.31	3.72	3.97	3.84

NSP, non-starch polysaccharide; IDCP, ileal digestible crude protein, assessed through *in vitro* simulation; NDF, neutral detergent fiber; ADF, acid detergent fiber; ADL, acid detergent lignin; VAF, volatile fatty acid.

1) Con, control treatment; CL, cassava leaves meal treatment; TF, tofu by-product treatment; CR, cassava root residue treatment.

2) Either cassava leaves meal, tofu by-product or cassava root residue.

3) Finely ground cassava root (Feedipedia.org).

4) L-lysine HCL containing 78.8% lysine.

5) ND, not determined due to unreliable NDF analysis of cassava root (see Table 1).

**Table 3:** Feed ingredients and chemical composition of experimental diets in the second period (50 to 80 kg of body weight)

Items	Diet1)			

	Con	CL	TF	CR
Ingredient (%)				
Test ingredient2)	-	7.49	3.99	9.26
Finely ground corn	35.0	28.4	25.0	25.0
Rice bran	33.1	15.0	21.6	10.8
Cassava powder3)	17.6	33.1	32.8	35.1
Soybean meal	9.00	13.0	11.6	12.9
Fish meal	5.30	3.00	5.00	6.90
Soybean oil	1.90	1.80	2.00	2.00
Chemical composition				
DM (%)	91.7	93.4	91.4	91.4
Crude protein (%)	15.2	15.1	15.3	14.7
Metabolisable energy (MJ/kg)	15.2	15.2	15.2	15.2
Ether extract (%)	7.20	5.00	5.10	4.70
Ash (%)	4.60	4.60	4.50	4.30
NSP (%)	10.0	15.0	15.0	15.0
IDCP (g/kg)	121	121	123	123
NDF (%)	19.8	18.8	19.1	ND4)
ADF (%)	6.86	8.66	8.25	8.79
ADL (%)	1.54	2.15	1.53	1.52
Starch (%)	48.9	46.5	46.3	48.2
Insoluble fiber (%)	18.3	17.4	18.0	16.1
Soluble fiber (%)	2.72	2.80	2.92	3.06
Total dietary fiber (%)	21.0	20.2	20.9	19.2
Total VFA (μmol/g)	3.67	4.12	4.19	4.23

DM, dry matter; NSP, non-starch polysaccharide; IDCP, ileal digestible crude protein, assessed through *in vitro* simulation; NDF, neutral detergent fiber; ADF, acid detergent fiber; ADL, acid detergent lignin; VAF, volatile fatty acid.

1) Con, control treatment; CL, cassava leaves meal treatment; TF, tofu by-product treatment; CR, cassava root residue treatment.

2) Either cassava leaves meal, tofu by-product or cassava root residue.

3) Finely ground cassava root (Feedipedia.org).

4) ND, not determined due to unreliable NDF analysis of cassava root (see Table 1).

**Table 4:** Feed intake and pig performance when feeding diets including one of the three fiber sources as compared with a control diet and assessed during two experimental periods (from about 28 kg until 50 kg of body weight and from 50 kg to 80 kg of body weight)

Variable	Diet1)				SEM	p-value

	Con	CL	TF	CR		
First period (n = 10)						
Initial body weight (kg)	28.7	28.7	28.8	28.3	0.60	0.95
Final body weight (kg)	51.3	50.3	54.3	52.9	1.40	0.16
Feed intake (kg feed/d)	1.89	1.93	2.12(*)	1.96	0.07	0.14
NSP intake (g/d)	189	289**	318**	294**	9.87	0.00
ADG (g/d)	727	695	827	794	33.3	0.03
FCR (kg feed/kg BW)	2.60	2.79(*)	2.57	2.48	0.06	0.01
Second period (n = 6)						
Initial body weight (kg)	51.4	49.6	54.4	53.1	1.36	0.10
Final body weight (kg)	78.3	76.2	84.3	87.8*	2.39	0.01
Feed intake (kg feed/d)	2.54	2.36	2.59	2.72	0.12	0.27
NSP intake (g/d)	254	354**	389**	408**	18.2	0.00
ADG (g/d)	725	718	806	937*	50.3	0.02
FCR (kg feed/kg BW)	3.52	3.33	3.23	2.95*	0.14	0.07

SEM, standard error of the mean; NSP, non-starch polysaccharide; ADG, average daily gain; FCR, feed conversion ratio; BW, body weight.

1) Con, control treatment; CL, cassava leaves meal treatment; TF, tofu by-product treatment; CR, cassava root residue treatment.

* Significantly different from the control treatment *(p<0.05),

** (p<0.01),

(*) (0.05<p<0.1).

**Table 5:** Total tract apparent digestibility (g/g) of various chemical fractions for a control diet and three experimental diets including one of the three fiber-rich sources1)

Variable	Diet2)				SEM	p-value

	Con	CL	TF	CR		
Dry matter	0.87	0.85	0.87	0.89**	0.006	0.007
Organic matter	0.90	0.87*	0.90	0.91	0.005	0.004
Crude protein	0.83	0.78(*)	0.82	0.84	0.009	0.005
Ether extract	0.81	0.69*	0.75	0.85	0.025	0.004

SEM, standard error of the mean.

1) Digestibilities were determined in pigs of about 50 kg of body weight (n = 4).

2) Con, control treatment; CL, cassava leaves meal treatment; TF, tofu by-product treatment; CR, cassava root residue treatment.

* Significantly different from the control treatment * (p<0.05),

** (p<0.01),

(*) (0.05<p<0.1).

**Table 6:** Nitrogen balance, fecal concentration of purine bases and volatile fatty acids of pigs of about 50 kg of body weight fed diets including either one of the three fiber-rich sources (n = 4)

Variable	Diet1)				SEM	p value

	Con	CL	TF	CR		
N intake (g/d)	50.7	49.1	49.7	49.8	2.10	0.95
N-feces (g/d)	8.64	10.7	9.03	8.04	0.70	0.09
N-Urine (g/d)	18.2	17.8	16.0	17.5	1.56	0.78
Total N excretion (% NI)	52.7	58.1	50.4	51.2	3.04	0.32
N-urine (% NE)	67.8	62.5	63.7	68.1	1.90	0.14
N retention (g/d)	23.9	20.6	24.6	24.3	1.70	0.36
Urinary N/fecal N	2.5	1.9	1.9	2.4	0.20	0.09
Purine bases (g/g DM)	1.55	1.47	1.75	1.82	0.11	0.15
Purine bases N/fecal N	0.23	0.19	0.24	0.24	0.01	0.10
Fecal VFA (g/kg DM)						
Acetate	23.8	27.4	30.6	25.3	2.32	0.24
Propionate	9.30	9.41	11.5	9.74	0.87	0.31
Butyrate	4.39	5.85	7.79*	6.22	0.77	0.06
Iso-butyrate	1.23	1.33	1.49	1.90**	0.10	0.003
Iso-valerate	2.22	2.49	2.62	3.41**	0.19	0.004
Valerate	1.89	1.75	2.69(*)	2.22	0.24	0.07
Caproate	0.33	0.39	0.61	0.24(*)	0.08	0.03
Total VFA	43.1	48.6	57.3(*)	49.0	4.10	0.17

SEM, standard error of the mean; NI, nitrogen intake; NE, nitrogen excretion; DM, dry matter; VFA, volatile fatty acid.

1) Con, control treatment; CL, cassava leaves meal treatment; TF, tofu by-product treatment; CR, cassava root residue treatment.

* Significantly different from the Control treatment * (p<0.05),

** (p<0.01),

(*) (0.05<p<0.1).
